# Aspiration Thrombectomy Using Inari FlowTriever System for Inferior Vena Cava Tumor Thrombus: A Case Report

**DOI:** 10.7759/cureus.58380

**Published:** 2024-04-16

**Authors:** Farman Ali, Khurram Arshad, Aman Ullah, Rabia Latif, Muhammad Bilal, FNU Zafrullah

**Affiliations:** 1 Medicine, St. John Hospital and Medical Center, Detroit, USA; 2 Internal Medicine, Corewell Health East Dearborn, Dearborn, USA; 3 Internal Medicine, St. Joseph Mercy Oakland Hospital, Pontiac, USA; 4 Internal Medicine, Mclaren Flint, Flint, USA; 5 Internal Medicine, Merit Health Wesley, Hattiesburg, USA; 6 Interventional Cardiology, Ascension Borgess Hospital, Kalamazoo, USA

**Keywords:** inari, thrombolytics, clottriever, flowtriever device, aspiration thrombectomy, pulmonary embolism (pe), inferior vena cava tumor thrombus

## Abstract

Pharmacomechanical therapy and catheter-directed thrombolysis are potent treatments for venous thromboembolism. However, limited data exist regarding the management of thrombi in the inferior vena cava (IVC). IVC thrombus resulting from tumors is a particularly uncommon condition. Managing IVC tumor thrombi poses even greater challenges, as conventional therapies such as systemic anticoagulation and thrombolysis are often ineffective. In this report, we present the case of a 73-year-old male with an inferior vena cava tumor thrombus successfully managed through aspiration thrombectomy utilizing the Inari FlowTriever system.

## Introduction

The FlowTriever System and the ClotTriever System, manufactured by Inari Medical (Irvine, California, USA), are mechanical thrombectomy devices designed for treating clotting disorders such as pulmonary embolism (PE) and deep vein thrombosis (DVT [1,2]. FlowTriever is a mechanical thrombectomy device that extracts thrombi from blood vessels and is primarily used to treat PE [1]. The ClotTriever System is a disposable endovascular apparatus that eliminates thrombi from peripheral veins [2]. The Inari FlowTriever system was the first device approved by the FDA in 2018 for mechanical thrombectomy of pulmonary embolism (PE) [3]. The device consists of a large-bore aspiration catheter (LBAC) and a self-expanding nitinol mesh that forms three rings to disrupt the thrombus. In the multicenter prospective FlowTriever Pulmonary Embolectomy Clinical Study (FLARE) trial, the safety and efficacy of the FlowTriever system for the treatment of intermediate-risk PE was demonstrated, and significant improvement in right ventricular function was observed, with fewer adverse events [4]. The potential uses of the LBAC devices outside the pulmonary system have not been well studied. Many reported cases and case series exist in the literature of using the Inari FlowTriever system to treat acute thrombus in large veins such as inferior vena cava (IVC), superior vena cava, and iliofemoral veins. Our case describes a novel application of the Inari FlowTriever system to treat an IVC tumor thrombus without significant complications.

## Case presentation

**Figure 1:**
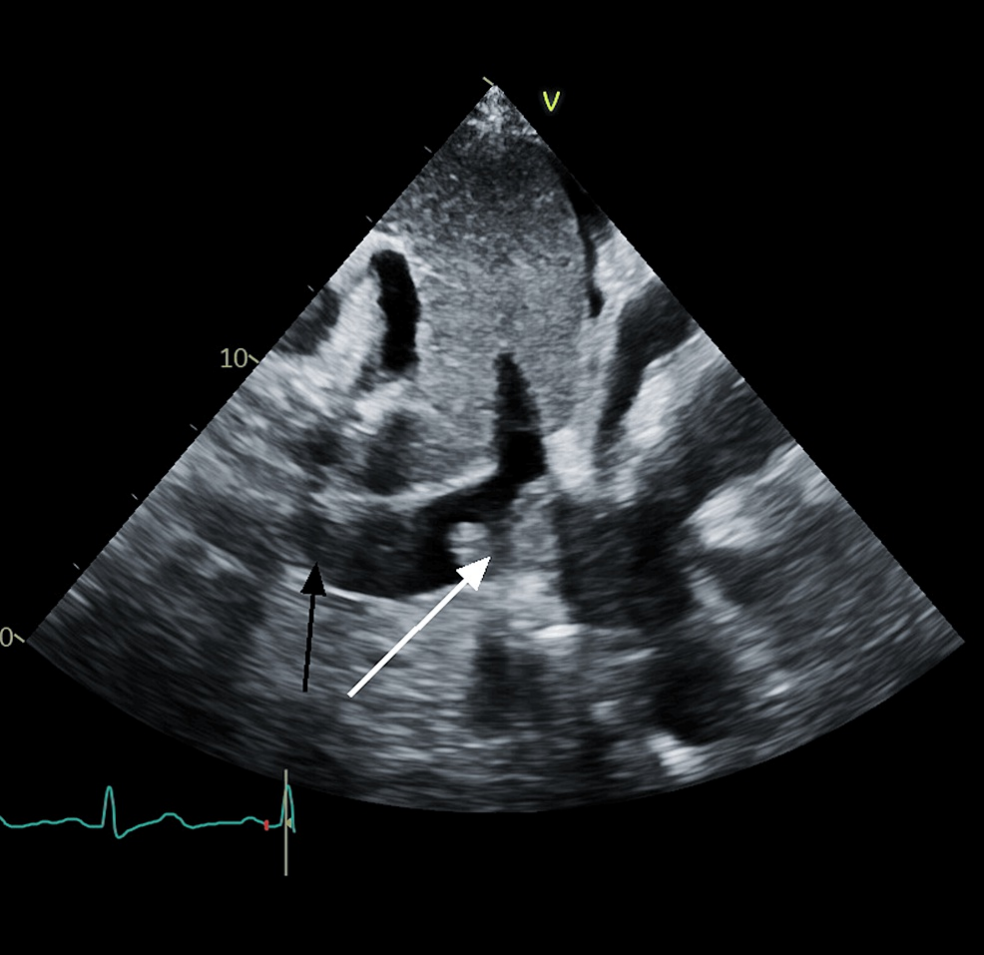
IVC thrombus on echocardiogram The black arrow indicates inferior vena cava (IVC), and the white arrow shows a large thrombus in IVC.

A 73-year-old male with a medical history significant for hyperlipidemia and coronary artery disease, previously treated with percutaneous angioplasty, presented to the emergency department (ED) complaining of worsening shortness of breath, orthopnea and a 14-pound weight gain over the past three weeks. Upon ED admission, vital signs were stable. Physical examination revealed significant bilateral lower extremity edema extending up to the knees. Chest X-ray demonstrated cardiomegaly and pleural effusions. Laboratory investigations revealed mild pancytopenia and an elevated brain natriuretic peptide (BNP) level of 1118 pg/ml. Further evaluation with echocardiography revealed a left ventricular ejection fraction (EF) of 55-60%, along with moderate pericardial effusion and a large thrombus in the IVC extending up to the cavoatrial junction (CAJ) (Figure 1). There was no thrombus in the right ventricle with normal function.

**Figure 2:**
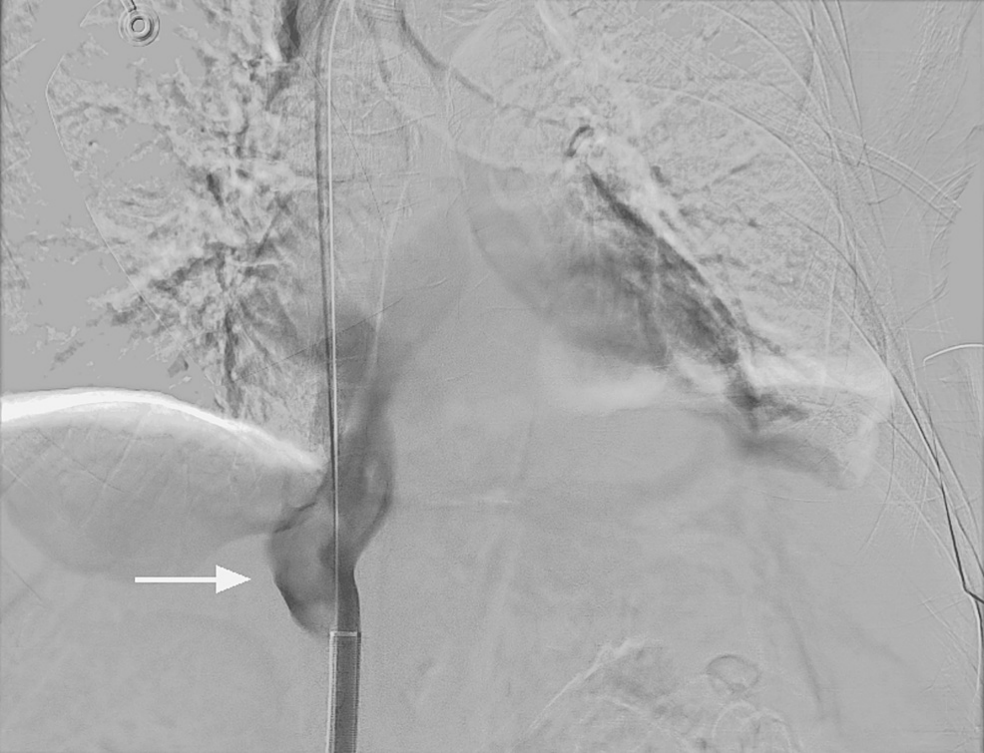
IVC venogram showing large IVC thrombus IVC: Inferior vena cava

The patient was started on a heparin drip for anticoagulation therapy targeting the IVC thrombus. The patient also underwent a CT abdomen as he was complaining of abdominal pain, which revealed a substantial mass in the right hepatic lobe measuring 8.4 x 9.1 x 9.6 cm. The patient's alpha fetor protein (AFP) level was 2 ng/ml (normal value 0.0-9.0 ng/ml). A liver mass biopsy was performed that came back positive for adenocarcinoma with a possible primary tumor of pancreaticobiliary origin, given a large liver mass. Due to the significant size of the thrombus, there was a considerable risk of embolization, so it was decided to proceed with thrombectomy. IVC venography demonstrated a large filling defect consistent with a thrombus in the IVC (Figure 2).

**Figure 3:**
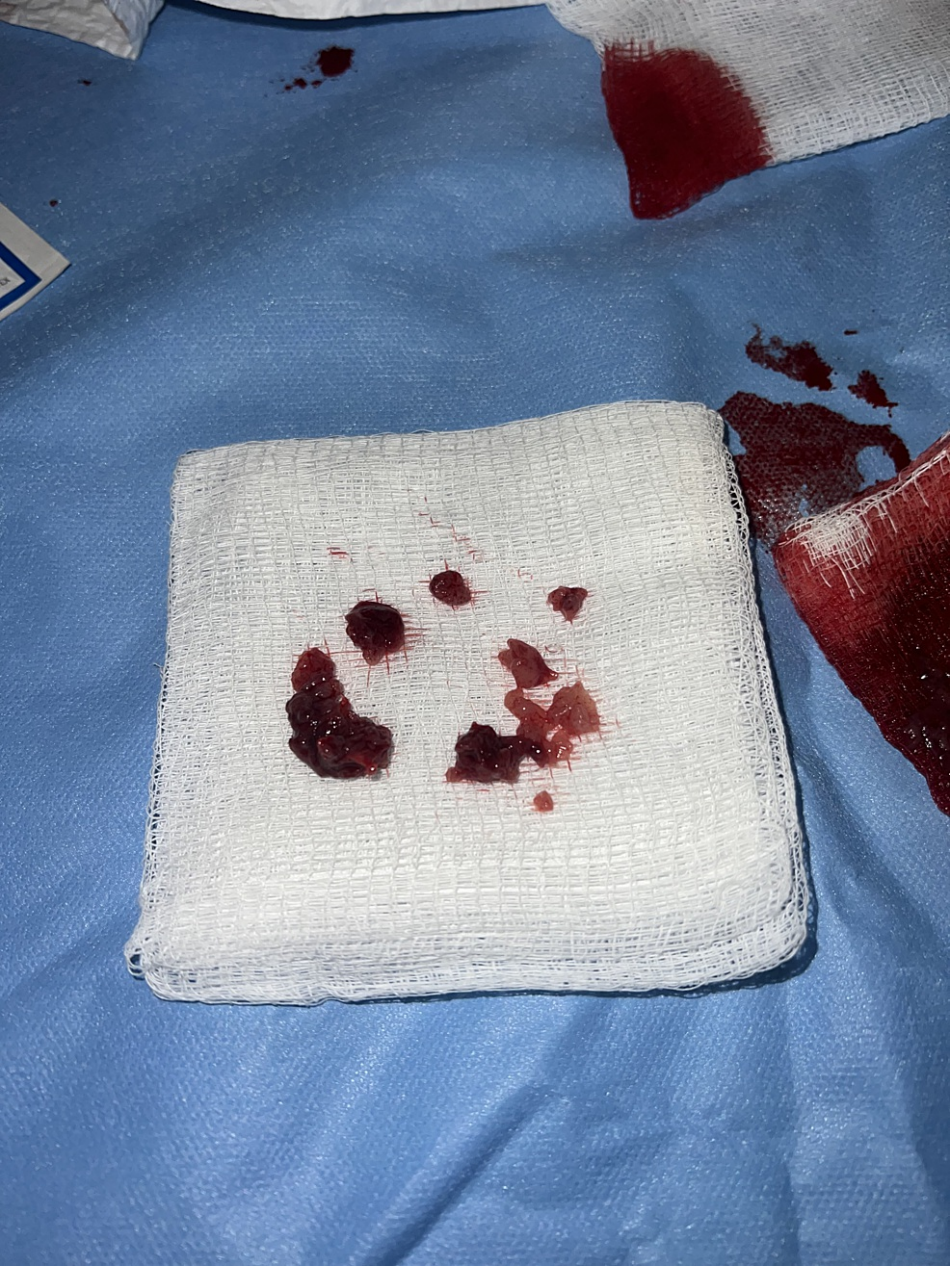
Specimen from IVC showing thrombus removed via Inari device IVC: Inferior vena cava Inari FlowTriever (Inari Medical, Irvine, California).

Under ultrasound guidance, the right internal jugular vein was accessed, and the Inari FlowTriever XL Disc was introduced, deploying a single disc near the confluence of the internal jugular and subclavian veins (CAJ) to function as a filter, mitigating the risk of pulmonary embolization. Subsequently, aspiration thrombectomy was conducted via the right femoral vein using the Inari FlowTriever catheter, which was then advanced into the inferior vena cava (IVC). Friable thrombotic material was extracted during aspiration. Pathological examination of the specimen confirmed the presence of adenocarcinoma (Figure 3).

During the hospital course, the patient's clinical condition continued to deteriorate, and the patient was discharged home with hospice care. In this case, the Inari FlowTriever device was used to remove the tumor clot from IVC.

## Discussion

Inferior vena cava (IVC) thrombosis is linked with the pathological and clinical manifestations of deep vein thrombosis (DVT). It is an under-recognized condition, as it is not routinely identified as a separate entity [5]. The lifetime incidence of DVT is 0.1%, with approximately 4-15% of patients with confirmed DVT experiencing IVC thrombosis [6,7]. Subsequently, IVC thrombosis poses a diagnostic and therapeutic challenge for clinicians as it is rarely described as a separate entity. This case details the successful removal of an IVC tumor thrombus using the FlowTriever system by Inari Medical. Managing IVC thrombus is challenging and varies depending on its underlying etiology. Treatment options are long-term anticoagulation, systemic thrombolysis, endovascular intervention involving catheter-directed thrombolysis (CDT) with or without mechanical thrombectomy, and surgical interventions. Surgical options are bypass, replacement, and ligation. These are rarely used due to high associated morbidity and mortality [8].

Medical therapy is ineffective, as less than 10% of patients achieve clot lysis in 10 days, and approximately 40% show evidence of thrombus propagation [9]. Systemic thrombolysis does not always result in rapid thrombus resolution and carries a higher risk of bleeding [9]. Conventional medical treatments, CDT, and systemic thrombolysis demonstrate inefficacy against tumor thrombus. A study by CaVenT demonstrated that catheter-directed thrombolysis (CDT), whether used alone or combined with mechanical thrombectomy, is a safe and efficient approach for addressing iliofemoral thrombus [10]. There is limited data regarding the effectiveness of CDT or mechanical thrombectomy for IVC thrombus treatment, as this condition is not frequently seen. Pharmaco-mechanical therapy has shown greater efficacy compared to CDT alone, particularly in terms of clot lysis and venous recanalization [8]. Regarding tumor thrombus management, treatment is even more complicated as tumor thrombuses are not very responsive to anticoagulation and thrombolytic therapy.

Using the Flow Triever technology carries the potential for clot embolism. A prior study suggested deploying a temporary embolic protection device upstream of the occlusion to protect the lungs [11]. Apart from the Inari device, other available devices for aspiration thrombectomy include the AngioJet, the Arrow-Trerotola, and the AngioVac. The AngioVac has the closest resemblance to the FlowTriever device as it employs large-bore aspiration of thrombus in the central veins. However, the AngioVac requires extracorporeal bypass and is indicated for venous thrombectomy. As there is limited data regarding comparative effectiveness and the lowest risk profile, the interventionalist often decides based on familiarity and comfort with these devices.

## Conclusions

There are no clear guidelines regarding IVC thrombus management. Pharmaco-mechanical therapy is preferred compared to catheter-directed thrombolysis. When an IVC thrombus is caused by a tumor, it presents an even bigger challenge for clinicians. Aspiration thrombectomy with the Inari FlowTriever system provides a novel option for tumor thrombi removal with minimum bleeding risk and less likelihood of pulmonary embolization. Many successful reported cases of treating tumor thrombi with this device exist, but more studies are needed to assess the long-term efficacy and risk profile of using these newer devices.
